# 2-Amino­pyrimidinium dihydrogen phosphate monohydrate

**DOI:** 10.1107/S1600536811010658

**Published:** 2011-03-26

**Authors:** Houda Marouani, Salem S. Al-Deyab, Mohamed Rzaigui

**Affiliations:** a Laboratoire de Chimie des Matériaux, Faculté des Sciences de Bizerte, 7021 Zarzouna Bizerte, Tunisia; b Petrochemical Research Chair, College of Science, King Saud University, Riadh, Saudi Arabia

## Abstract

In the title compound, C_4_H_6_N_3_^+^·H_2_O_4_P^−^·H_2_O, the pyrimidin­ium ring is essentially planar, with an r.m.s. deviation of 0.0016 Å. In the structure, pairs of symmetry-related anions are connected into centrosymmetric clusters *via* strong O—H⋯O hydrogen bonds forming six-membered rings with an *R*_2_^2^(6) motif. These clusters are inter­connected *via* water mol­ecules through O*W*—H⋯O hydrogen bonds, building an infinite layer parallel to the *ab* plane. Moreover, infinite chains of 2-amino­pyrimidinium cations spread along the *a*-axis direction. These chains are connected to the inorganic layer through N—H⋯O, C—H⋯O and C—H⋯N hydrogen bonds, which, together with electrostatic and van der Waals inter­actions, contribute to the cohesion and stability of the network in the crystal structure.

## Related literature

For the biological activity of amino­pyrimidinium derivatives, see: Grier *et al.* (1980 ▶); Gueiffier *et al.* (1996 ▶); Rival *et al.* (1991 ▶); Li *et al.* (2009 ▶). For related structures, see: Cheng *et al.* (2010 ▶); Narayana *et al.* (2008 ▶). For graph-set notation of hydrogen bonding, see: Bernstein *et al.* (1995 ▶).
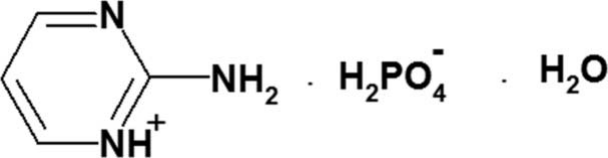


## Experimental

### 

#### Crystal data

C_4_H_6_N_3_^+^·H_2_PO_4_^−^·H_2_O
*M* *_r_* = 211.12
Triclinic, 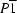

*a* = 6.212 (3) Å
*b* = 8.600 (4) Å
*c* = 9.462 (2) Å
α = 109.56 (3)°
β = 106.38 (2)°
γ = 95.50 (2)°
*V* = 446.7 (3) Å^3^
*Z* = 2
Ag *K*α radiation
λ = 0.56083 Å
μ = 0.17 mm^−1^
*T* = 293 K
0.40 × 0.25 × 0.20 mm

#### Data collection

Enraf–Nonius CAD-4 diffractometer
6572 measured reflections
4373 independent reflections
3189 reflections with *I* > 2σ(*I*)
*R*_int_ = 0.018
2 standard reflections every 120 min  intensity decay: 3%

#### Refinement

*R*[*F* ^2^ > 2σ(*F* ^2^)] = 0.042
*wR*(*F* ^2^) = 0.124
*S* = 1.03
4373 reflections
158 parameters
3 restraints
All H-atom parameters refined
Δρ_max_ = 0.68 e Å^−3^
Δρ_min_ = −0.42 e Å^−3^

### 

Data collection: *CAD-4 EXPRESS* (Enraf–Nonius, 1994) ▶; cell refinement: *CAD-4 EXPRESS* ▶; data reduction: *XCAD4* (Harms & Wocadlo, 1995) ▶; program(s) used to solve structure: *SHELXS86* (Sheldrick, 2008 ▶); program(s) used to refine structure: *SHELXL97* (Sheldrick, 2008 ▶); molecular graphics: *ORTEP-3 for Windows* (Farrugia, 1997 ▶); software used to prepare material for publication: *WinGX* (Farrugia, 1999 ▶).

## Supplementary Material

Crystal structure: contains datablocks I, global. DOI: 10.1107/S1600536811010658/pv2399sup1.cif

Structure factors: contains datablocks I. DOI: 10.1107/S1600536811010658/pv2399Isup2.hkl

Additional supplementary materials:  crystallographic information; 3D view; checkCIF report

## Figures and Tables

**Table 1:** Hydrogen-bond geometry (Å, °)

*D*—H⋯*A*	*D*—H	H⋯*A*	*D*⋯*A*	*D*—H⋯*A*
O1—H1*O*⋯O4^i^	0.83 (3)	1.80 (3)	2.6372 (18)	179 (2)
O2—H2*O*⋯O*W*	0.82 (3)	1.80 (3)	2.6132 (18)	175 (2)
O*W*—H1*W*⋯O3^ii^	0.84 (1)	1.96 (1)	2.7882 (17)	166 (2)
O*W*—H2*W*⋯O4^iii^	0.83 (1)	1.95 (1)	2.7843 (16)	177 (2)
N1—H1⋯O4^iv^	0.83 (2)	2.08 (2)	2.9070 (18)	177 (2)
N1—H2⋯O3^v^	0.89 (2)	2.00 (2)	2.873 (2)	166 (2)
N2—H3⋯O3^iv^	0.87 (2)	1.78 (2)	2.6535 (16)	175 (2)
C2—H4⋯N3^iii^	0.91 (2)	2.62 (2)	3.513 (2)	169 (2)
C3—H5⋯O*W*	0.95 (2)	2.56 (2)	3.477 (2)	164 (2)

Symmetry codes: (i) 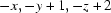; (ii) 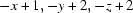; (iii) 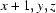; (iv) 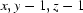; (v) 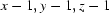.

**Fig. 1.**
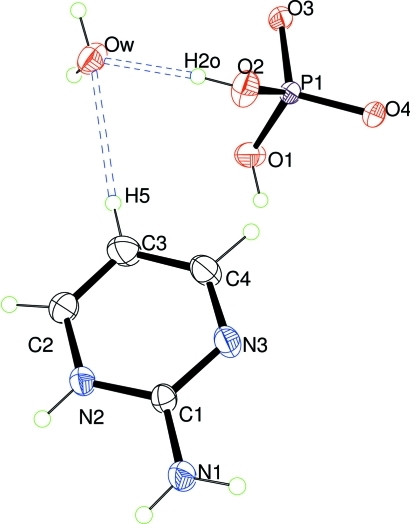
An ORTEP view of of the title compound with the atom-labelling scheme. Displacement ellipsoids are drawn at the 30% probability level. H atoms are represented by small spheres of arbitrary radii.

**Fig. 2.**
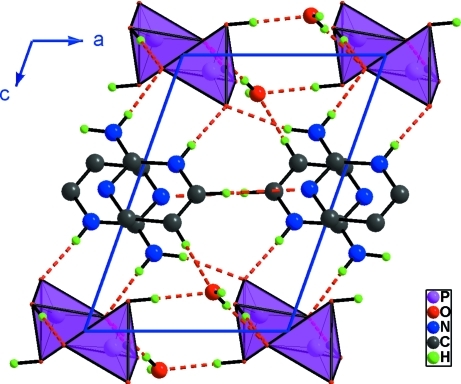
Projection of (I) along the b axis.

## supplementary crystallographic information

### Comment

Aminopyrimidinium salts are highly active antimicrobial agents with a low acute mammalian toxicity (Grier *et al.*, 1980). In addition, pyrimidine derivatives possess considerable biological activity and have been widely used in medicinal applications as antiviral agents (Gueiffier *et al.*, 1996), antifungal agents (Rival *et al.*, 1991). Moreover, imidazolidinonyl aminopyrimidine compounds have been investigated for the treatment of cancer (Li *et al.*, 2009). In order to search for new materials for these applications, we have attempted to combine pyrimidine derivatives with phosphate species. In this paper we report the preparation and the crystal structure of the title compound, (I).

The asymmetric unit of (I) contains a H_2_PO_4_^-^ anion, a 2-aminopyrimidinium cation and a water molecule (Fig. 1). The pyrimidinium ring is essentially planar with an rms deviation of 0.0016 Å. The interatomic bond lengths and angles in (I) do not show significant deviation from those reported in related 2-aminopyrimidinium salts (Narayana, *et al.*, 2008; Cheng, *et al.*, 2010).

In the structure, pairs of symmetry-related anions are connected into centrosymmetric clusters *via* strong O—H···O hydrogen bonds forming six-membred rings which may be described as *R*_2_^2^(6) motif in the graph-set notation (Bernstein *et al.*, 1995). These clusters are interconnected *via* water molecules through OW—H···O hydrogen bonds to build an infinite layer parallel to the *ab* plane. Moreover, infinite chains of 2-aminopyrimidinium cations spread along the *a* direction. These chains are connected to the inorganic layer through H-bonds: N—H···O, C—H···O and C—H···N (Tab. 1 & Fig. 2).

### Experimental

A small quantity of H_3_PO_4_ (3 mmol) was added dropwise to a solution of 2-aminopyrimidine (3 mmol in 20 ml water). A precipitate was formed which was dissolved in water (20 ml) and the solution was allowed to evaporate slowly at room temperature until the formation of colorless prismatic crystals with dimensions suitable for a crystallographic study.

### Refinement

All H atoms were located in difference Fourier maps and were allowed to refine with isotropic displacement parameters *U*_iso_. The H-atoms of water molecule were refined with a distance restraint of O—H = 0.85 (1) Å.

### Crystal data

**Table:**

C_4_H_6_N_3_^+^·H_2_PO_4_^−^·H_2_O	*Z* = 2
*M**_r_* = 211.12	*F*(000) = 220
Triclinic, *P*1	*D*_x_ = 1.570 Mg m^−^^3^
Hall symbol: -P 1	Ag *K*α radiation, λ = 0.56083 Å
*a* = 6.212 (3) Å	Cell parameters from 25 reflections
*b* = 8.600 (4) Å	θ = 9–11°
*c* = 9.462 (2) Å	µ = 0.17 mm^−^^1^
α = 109.56 (3)°	*T* = 293 K
β = 106.38 (2)°	Prism, colorless
γ = 95.50 (2)°	0.40 × 0.25 × 0.20 mm
*V* = 446.7 (3) Å^3^	

### Data collection

**Table:**

Enraf–Nonius CAD-4 diffractometer	*R*_int_ = 0.018
Radiation source: fine-focus sealed tube	θ_max_ = 28.0°, θ_min_ = 2.0°
graphite	*h* = −10→10
non–profiled ω scans	*k* = −14→14
6572 measured reflections	*l* = −5→15
4373 independent reflections	2 standard reflections every 120 min
3189 reflections with *I* > 2σ(*I*)	intensity decay: 3%

### Refinement

**Table:**

Refinement on *F*^2^	Primary atom site location: structure-invariant direct methods
Least-squares matrix: full	Secondary atom site location: difference Fourier map
*R*[*F*^2^ > 2σ(*F*^2^)] = 0.042	Hydrogen site location: inferred from neighbouring sites
*wR*(*F*^2^) = 0.124	All H-atom parameters refined
*S* = 1.03	*w* = 1/[σ^2^(*F*_o_^2^) + (0.0745*P*)^2^ + 0.0087*P*] where *P* = (*F*_o_^2^ + 2*F*_c_^2^)/3
4373 reflections	(Δ/σ)_max_ = 0.002
158 parameters	Δρ_max_ = 0.68 e Å^−^^3^
3 restraints	Δρ_min_ = −0.42 e Å^−^^3^

### Special details

**Table:**

Geometry. All e.s.d.'s (except the e.s.d. in the dihedral angle between two l.s. planes) are estimated using the full covariance matrix. The cell e.s.d.'s are taken into account individually in the estimation of e.s.d.'s in distances, angles and torsion angles; correlations between e.s.d.'s in cell parameters are only used when they are defined by crystal symmetry. An approximate (isotropic) treatment of cell e.s.d.'s is used for estimating e.s.d.'s involving l.s. planes.
Refinement. Refinement of *F*^2^ against ALL reflections. The weighted *R*-factor *wR* and goodness of fit *S* are based on *F*^2^, conventional *R*-factors *R* are based on *F*, with *F* set to zero for negative *F*^2^. The threshold expression of *F*^2^ > σ(*F*^2^) is used only for calculating *R*-factors(gt) *etc*. and is not relevant to the choice of reflections for refinement. *R*-factors based on *F*^2^ are statistically about twice as large as those based on *F*, and *R*- factors based on ALL data will be even larger.

### Fractional atomic coordinates and isotropic or equivalent isotropic displacement parameters (Å^2^)

**Table:**

	*x*	*y*	*z*	*U*_iso_*/*U*_eq_	
P1	0.16874 (4)	0.74641 (3)	1.06016 (4)	0.02665 (8)	
O3	0.30367 (14)	0.91480 (10)	1.18597 (11)	0.03404 (18)	
O4	−0.07714 (13)	0.70387 (10)	1.05045 (11)	0.03351 (18)	
O1	0.30374 (15)	0.61277 (12)	1.09639 (14)	0.0442 (3)	
O2	0.16298 (17)	0.73861 (14)	0.89151 (12)	0.0433 (2)	
OW	0.56449 (16)	0.79100 (13)	0.85902 (13)	0.0406 (2)	
N2	0.15897 (16)	0.12980 (13)	0.39597 (13)	0.03242 (19)	
N3	−0.14127 (17)	0.21993 (14)	0.48619 (13)	0.0348 (2)	
N1	−0.20502 (17)	−0.03637 (13)	0.28151 (13)	0.0352 (2)	
C1	−0.06315 (18)	0.10520 (14)	0.38722 (13)	0.02783 (19)	
C2	0.3116 (2)	0.27019 (17)	0.50499 (18)	0.0419 (3)	
C4	0.0099 (2)	0.35703 (17)	0.59250 (17)	0.0401 (3)	
C3	0.2421 (3)	0.38927 (18)	0.60766 (19)	0.0467 (3)	
H1W	0.608 (3)	0.8881 (14)	0.862 (2)	0.052 (5)*	
H1	−0.164 (3)	−0.111 (2)	0.218 (2)	0.049 (5)*	
H1O	0.232 (4)	0.513 (3)	1.051 (3)	0.075 (7)*	
H3	0.201 (3)	0.054 (2)	0.327 (2)	0.049 (5)*	
H6	−0.051 (3)	0.439 (2)	0.662 (2)	0.044 (4)*	
H5	0.347 (4)	0.487 (3)	0.689 (3)	0.064 (6)*	
H2	−0.355 (4)	−0.047 (2)	0.269 (2)	0.053 (5)*	
H4	0.455 (4)	0.272 (3)	0.499 (2)	0.060 (6)*	
H2W	0.668 (3)	0.761 (2)	0.916 (2)	0.065 (6)*	
H2O	0.289 (4)	0.761 (3)	0.883 (3)	0.078 (7)*	

### Atomic displacement parameters (Å^2^)

**Table:**

	*U*^11^	*U*^22^	*U*^33^	*U*^12^	*U*^13^	*U*^23^
P1	0.01938 (11)	0.02235 (12)	0.03390 (15)	0.00234 (8)	0.00888 (9)	0.00599 (9)
O3	0.0271 (3)	0.0257 (3)	0.0406 (5)	−0.0005 (3)	0.0135 (3)	0.0018 (3)
O4	0.0216 (3)	0.0284 (3)	0.0472 (5)	0.0027 (3)	0.0124 (3)	0.0102 (3)
O1	0.0261 (4)	0.0291 (4)	0.0649 (7)	0.0059 (3)	0.0023 (4)	0.0132 (4)
O2	0.0328 (4)	0.0556 (6)	0.0366 (5)	0.0013 (4)	0.0123 (4)	0.0130 (4)
OW	0.0297 (4)	0.0428 (5)	0.0520 (6)	0.0031 (3)	0.0113 (4)	0.0244 (4)
N2	0.0256 (4)	0.0323 (4)	0.0358 (5)	0.0064 (3)	0.0124 (4)	0.0068 (4)
N3	0.0295 (4)	0.0376 (5)	0.0353 (5)	0.0116 (4)	0.0132 (4)	0.0080 (4)
N1	0.0256 (4)	0.0340 (5)	0.0374 (5)	0.0050 (3)	0.0100 (4)	0.0038 (4)
C1	0.0244 (4)	0.0311 (4)	0.0284 (5)	0.0080 (3)	0.0093 (3)	0.0108 (4)
C2	0.0274 (5)	0.0376 (6)	0.0513 (8)	0.0022 (4)	0.0116 (5)	0.0079 (5)
C4	0.0398 (6)	0.0363 (5)	0.0388 (6)	0.0132 (5)	0.0132 (5)	0.0061 (5)
C3	0.0373 (6)	0.0356 (6)	0.0484 (8)	0.0031 (5)	0.0081 (5)	−0.0006 (5)

### Geometric parameters (Å, °)

**Table:**

P1—O4	1.5055 (11)	N3—C4	1.3207 (18)
P1—O3	1.5056 (12)	N3—C1	1.3473 (15)
P1—O1	1.5583 (11)	N1—C1	1.3194 (16)
P1—O2	1.5645 (11)	N1—H1	0.83 (2)
O1—H1O	0.83 (3)	N1—H2	0.89 (2)
O2—H2O	0.82 (3)	C2—C3	1.354 (2)
OW—H1W	0.843 (9)	C2—H4	0.91 (2)
OW—H2W	0.834 (9)	C4—C3	1.399 (2)
N2—C2	1.3473 (17)	C4—H6	0.98 (2)
N2—C1	1.3503 (15)	C3—H5	0.95 (2)
N2—H3	0.87 (2)		
			
O4—P1—O3	114.86 (5)	C1—N1—H2	118.5 (12)
O4—P1—O1	111.37 (6)	H1—N1—H2	118.3 (17)
O3—P1—O1	106.04 (6)	N1—C1—N3	119.50 (10)
O4—P1—O2	106.56 (6)	N1—C1—N2	118.97 (10)
O3—P1—O2	110.54 (6)	N3—C1—N2	121.51 (10)
O1—P1—O2	107.27 (7)	N2—C2—C3	119.76 (12)
P1—O1—H1O	115.6 (18)	N2—C2—H4	112.8 (13)
P1—O2—H2O	115.0 (18)	C3—C2—H4	127.4 (13)
H1W—OW—H2W	112.2 (18)	N3—C4—C3	124.26 (12)
C2—N2—C1	121.10 (11)	N3—C4—H6	115.4 (10)
C2—N2—H3	120.4 (13)	C3—C4—H6	120.3 (10)
C1—N2—H3	118.5 (13)	C2—C3—C4	116.54 (13)
C4—N3—C1	116.83 (11)	C2—C3—H5	121.6 (14)
C1—N1—H1	122.7 (13)	C4—C3—H5	121.8 (13)

### Hydrogen-bond geometry (Å, °)

**Table:**

*D*—H···*A*	*D*—H	H···*A*	*D*···*A*	*D*—H···*A*
O1—H1O···O4^i^	0.83 (3)	1.80 (3)	2.6372 (18)	179 (2)
O2—H2O···OW	0.82 (3)	1.80 (3)	2.6132 (18)	175 (2)
OW—H1W···O3^ii^	0.84 (1)	1.96 (1)	2.7882 (17)	166 (2)
OW—H2W···O4^iii^	0.83 (1)	1.95 (1)	2.7843 (16)	177 (2)
N1—H1···O4^iv^	0.83 (2)	2.08 (2)	2.9070 (18)	177 (2)
N1—H2···O3^v^	0.89 (2)	2.00 (2)	2.873 (2)	166 (2)
N2—H3···O3^iv^	0.87 (2)	1.78 (2)	2.6535 (16)	175 (2)
C2—H4···N3^iii^	0.91 (2)	2.62 (2)	3.513 (2)	169 (2)
C3—H5···OW	0.95 (2)	2.56 (2)	3.477 (2)	164 (2)

Symmetry codes: (i) −*x*, −*y*+1, −*z*+2; (ii) −*x*+1, −*y*+2, −*z*+2; (iii) *x*+1, *y*, *z*; (iv) *x*, *y*−1, *z*−1; (v) *x*−1, *y*−1, *z*−1.
